# Protein High-Force Pulling Simulations Yield Low-Force Results

**DOI:** 10.1371/journal.pone.0034781

**Published:** 2012-04-18

**Authors:** Seth Lichter, Benjamin Rafferty, Zachary Flohr, Ashlie Martini

**Affiliations:** 1 Department of Mechanical Engineering, Northwestern University, Evanston, Illinois, United States of America; 2 College of Engineering, Purdue University, West Lafayette, Indiana, United States of America; 3 School of Engineering, University of California Merced, Merced, California, United States of America; Université d'Evry val d'Essonne, France

## Abstract

All-atom explicit-solvent molecular dynamics simulations are used to pull with extremely large constant force (750–3000 pN) on three small proteins. The introduction of a nondimensional timescale permits direct comparison of unfolding across all forces. A crossover force of approximately 1100 pN divides unfolding dynamics into two regimes. At higher forces, residues sequentially unfold from the pulling end while maintaining the remainder of the protein force-free. Measurements of hydrodynamic viscous stresses are made easy by the high speeds of unfolding. Using an exact low-Reynolds-number scaling, these measurements can be extrapolated to provide, for the first time, an estimate of the hydrodynamic force on low-force unfolding. Below 1100 pN, but surprisingly still at extremely large applied force, intermediate states and cooperative unfoldings as seen at much lower forces are observed. The force-insensitive persistence of these structures indicates that decomposition into unfolded fragments requires a large fluctuation. This finding suggests how proteins are constructed to resist transient high force. The progression of 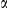 helix and 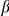 sheet unfolding is also found to be insensitive to force. The force-insensitivity of key aspects of unfolding opens the possibility that numerical simulations can be accelerated by high applied force while still maintaining critical features of unfolding.

## Introduction

Force-induced unfolding experiments have significant biological and medical importance because they provide insight into how proteins unfold. Proteins may experience both *in vivo* forces, such as those due to contact with cell walls, and man-made forces, such as shear imposed during production of protein-based drugs [1]–[3]. *In vivo* forces exerted by and on proteins range up to a few 100 pN [4], [5]. Protein functionality is highly dependent on its structure, so structural changes caused by external forcing can have significant and potentially dangerous consequences. Understanding how proteins respond to applied force can enable prediction of their force-induced functionality [6], [7].

It has been suggested that unfolding mechanisms might be force-dependent. Previous simulation-based constant high-force unfolding studies have identified critical transition forces that differentiate regimes of unfolding. Using a coarse-grained G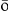-like molecular dynamics model, Szymczak and Cieplak studied the unfolding of ubiquitin (and integrin) [8], [9]. They found two types of unfolding scenarios separated by a critical value of the force. Though the unfolding times change significantly as applied force is varied, the sequence of secondary structure unfoldings depended only weakly on the magnitude of the force. Li, Kouza and Hu also carried out coarse-grained G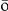 modeling of ubiquitin [10]. Their objective was to compare simulation with the constant-force AFM experiments of Fernandez and Li [11]. They identified the unfolding sequence and investigated the differences in unfolding when force was applied at the N-terminus alone, the C-terminus alone, or at both termini. They noted that contrary to thermally-induced denaturation, forced unfolding will unzip from the termini. As found in [8], they too find a critical force at which the unfolding times' dependence on force changes, rather abruptly, from exponential at low forces to a linear dependence. Luccioli et al. [12] carried out coarse-grained modeling of unfolding of a 46-residue 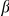 barrel protein. They found a critical force above which unfolding can be explained in terms of a force-induced drift, while at forces below critical, escape from the native state is thermally activated. (Note that in prior constant-force work, the critical transition forces found are much lower than the forces used in our work [8]–[10], [12]: we too find a crossover force, but it is due to a different mechanism.) Li and Marakov [13] determine the free-energy landscape under forces up to 250 pN applied in MD simulation to ubiquitin and streptococcal protein G IgG-binding domain III. For ubiquitin, the highest force nearly erases the free-energy minimum seen at lower forces. Unlike in [13], the crossover force found in this work, is defined in terms of changes in behavior–in folding times, variance, and appearance of intermediate states.

We report on protein response to extremely large forces, 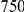 pN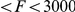 pN, with the goal of elucidating protein unfolding at both high and low forces. All-atom explicit solvent molecular dynamics is used to pull on three proteins–ubiquitin, barnase, and RNase H–while monitoring their unfolding. A crossover force of approximately 1100 pN divides unfolding dynamics into two regimes. At higher forces, residues sequentially unfold from the pulling end. The region below 1100 pN, but still at extremely large applied force 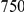 pN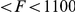 pN, is most interesting. Sequential unfolding is interrupted by intermediate states and cooperative unfolding. Residues identified in prior AFM studies as playing critical structural roles, unfold cooperatively even under the high forces used here [14]–[16]. The presence of cooperative structures at extremely high forces indicates that the landscape of downhill unfolding may possess interesting structure.

Atomic-force microscope-based techniques have been used to mechanically unfold proteins (see for example [11], [17], [18]); these experiments have been complemented by atomic-scale modeling such as that described in the previous paragraph. Relative to the typical millisecond to second unfolding times measured with the AFM and laser tweezers, atomic-scale numerical computation suffers from a relatively short achievable simulation time span. In order to completely unfold a protein within the available time, unfolding can be hastened by the application of a high force or by constraining the termini to move apart at a large constant speed [19]–[27]. While high force has been utilized to speed unfolding, few studies have focused on understanding the effects of high-forces *per se* [7], [28]. We show that unfolding at large forces preserve cooperative features. Hence, simulations at large applied forces have a place among numerical methodologies. The application of large force accelerates unfolding such that the entire unfolding process may be observed within a simulation while conserving cooperative events along the pathway.

The paper is organized as follows. The Results section is divided into subsections. In the subsections Intermediate States, and Cooperativity, the unfolding of specific residues and secondary structures at low force, using a variety of experimental and numerical methods, is compared with our results at high force. We find particular interactions, which contribute to cooperativity and intermediate states at low forces, are present under high-force unfolding. We then introduce a nondimensional timescale which permits comparison of unfolding at all forces: nondimensional results focus attention away from the *duration* of unfolding and onto the *sequence* of steps. In the subsection, 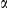 Helix and 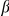 Strand Unfolding, we show that the number of unfolded residues follows a common trajectory as a function of nondimensional time within the range of forces below the crossover, with a different trajectory above the crossover force. In Unfolding Times and Length Scales, we find that at these extremely large applied forces, the usual energy scaling is not applicable, but rather a viscous scale appears. Here, and in the Coefficient of Variation subsection, we show that a crossover force divides a high-force regime, in which residues unfold one-by-one, from a lower-force regime of cooperative unfolding. Over the range of forces from 750–3000 pN, unfolding times vary by approximately a factor of ten. Finally, in the subsection Front Propagation Speed, we show that at the highest forces, unfolding is non-cooperative one-by-one, starting at the pulling end.

In the Discussion and Conclusions section, we consider the implications and potential utility of the findings. The high speeds of unfolding make viscous drag sizeable, allowing its measurement. A well-established scaling can be applied such that these measurements can be extrapolated to low-force unfolding. Below the crossover force, but still at extremely large applied forces, cooperativity persists, suggesting how proteins are constructed to resist high transient forces. A new scaled-time coordinate is used to show that the sequence of 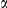 helix and 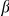 sheet unfolding is insensitive to force. The force-insensitivity of key aspects of unfolding opens the possibility that numerical simulations can be accelerated by high applied force while still maintaining critical features of unfolding.

Lastly, a Methods section describes the numerical technique, cell geometry, initial conditions, and the explicit solvent model.

## Results

### Cooperativity

**Figure 1:**
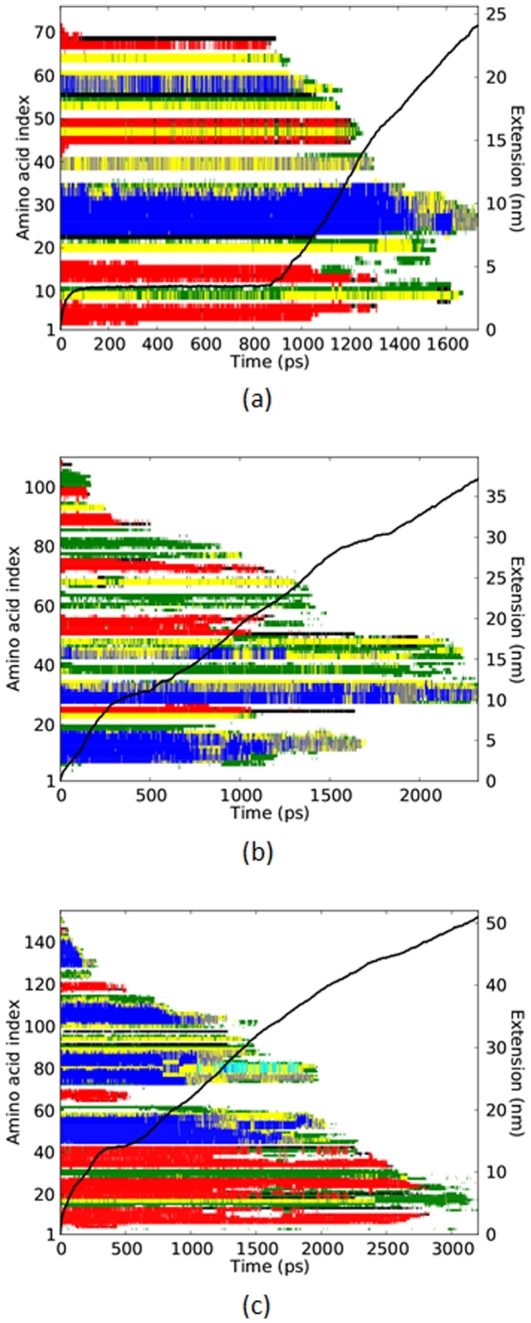
Secondary structures unfolding to coil. Each horizontal bar represents one residue. Colored residues (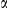 helix, blue; 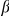 strand, red; turn, yellow; bend, green; 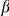-bridge, black; 3 helix, grey; 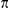 helix, cyan) are folded. The bar turns white at the time of transition to coil. The superimposed black curve is the end-to-end extension. (a) ubiquitin at 900 pN, (b) barnase at 750 pN, (c) RNase H at 900 pN.

The high-force simulations show–residue-by-residue–protein unfolding at a given applied force. Illustrative examples for each of the three proteins are shown in Fig. 1, where the colors indicate folded secondary structures and the black solid line shows the end-to-end extension as functions of time. Using this, as well as other data presented below, we investigate if unfolding behaviors observed experimentally at lower forces persist in simulations at high force.

At the forces used in this study, residues frequently unfold sequentially from the termini, especially from the pulling end. However, certain residues unfold out of sequence. For example, from Fig. 1a, we observe the following out-of-sequence residues unfolding near the start of the plateau of constant extension: residues 42–44 (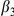) (simultaneously with 70 and 71 (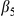)) at 20 ps, followed by residues 1 (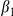) and 15 (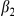) at 320 ps. In simulations at the much lower forces representative of AFM experiments [29], the separation of 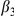 from 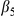 and 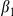 from 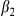 occur before or at the initiation of the plateau. Hence, superimposed on unfolding in sequence from the ends of ubiquitin, is the out-of-sequence unfolding of these key residues that confer stability to the long-lived plateau in extension. These out-of-sequence residues can be observed through surprisingly high applied forces, up to 2000 pN.

It can also be seen from Fig. 1a that the 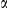 helices, the central blue region of residues 23–34, unfold last. The late unfolding of ubiquitin's 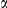 helices has been consistently observed in prior simulations at much lower forces [10], [29]–[32]. It can also be seen that the unfolding of residues 65–67 (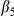) is concurrent with the ending time, at approximately 900 ps, of the plateau of constant extension. These residues are at one end of structure D, as identified by [30], which “plays a crucial role stabilizing role.” Though [30] was a simulation at much lower force levels, in range 100–200 pN, here too, at much larger forces, it appears that the critical role of structure D is preserved.

Barnase has a time span, 250–500 ps, during which the rate of extension is noticeably reduced, Fig. 1b. In prior simulations at much lower forces [33], the intermediate state was composed in part of core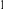 “with Ile88 being at the center,” and core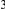 with “Leu63 and Leu89 being at the center.” (While core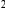 is “completely unfolded in the intermediate.”) As can be seen from Fig. 1b, we too find that 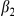 (residues 87–91) stabilizes the intermediate state, and the complete unfolding of 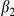 is approximately coincident with the end of the plateau interval at 500 ps.

For RNase H, pulse labeling hydrogen exchange identified a “core region” of stable structures consists of helix 1 (residues 44–58), helix 4 (residues 101–111) and 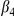 (residues 64–68) [34]. The unfolding of 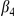 from 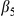 (residues 114–122) was one of the markers for the unfolding of the so-called 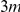 intermediate state [35]. Both studies cited establish a stabilizing role for 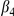 and/or its interaction with 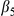. Our observations, up through an applied force of approximately 1000 pN, show a small plateau in the end-to-end extension whose unfolding (at approximately 500 ps, Fig. 1c) occurs concurrently with the unfolding of 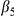 residues and with the out-of-sequence unfolding of 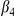.

### Intermediate states

**Figure 2:**
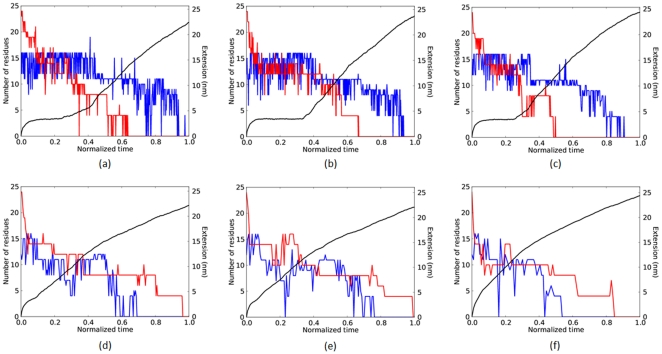
The number of folded 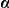-helix (blue) or 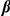-strand (red) residues in ubiquitin versus time nondimensionalized by the unfolding time, for different applied forces. The black line shows the end-to-end extension. (a) 875 pN, (b) 1000 pN, (c) 1250 pN, (d) 1500 pN, (e) 2000 pN, (f) 3000 pN.

Ubiquitin reveals an intermediate state on low-force unfolding [30], [32]. Evidence for the intermediate state persisting at high applied force is shown in Fig. 2 which shows typical realizations for the number of folded 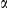-helical and 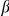-strand residues versus nondimensional time for ubiquitin. As shown by the solid black line, for forces of 1500 pN and above (Figs. 2a–2c), there is little or no evidence of a plateau in extension. For these three highest forces, 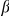 strands (shown in red) complete their unfolding after 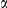 helix (shown in blue) unfolding. For lower forces, of 1250 pN and below (Figs. 2d–2f), the unfolding pathway is different. Now, 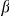 strands lag behind 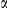 helices in completing their unfolding. And, noticeably, the pronounced plateau at a constant extension of approximately 3.3 nm is evidence of the longer-lived intermediate state.

In summary, specific features of unfolding–such as intermediate states, stabilizing residues and cooperative unfolding–persist at forces up to 1000 pN and above. We now show that the progression of 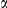 helix and 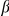 sheet unfolding can also be force insensitive.

### 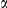 helix and 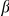 strand unfolding

Nondimensional time is defined as dimensional time divided by unfolding time, where unfolding times were determined as the first time at which all hydrogen-bonded secondary structures, as defined by the DSSP algorithm [36], unfold. The nondimensional time equalizes the duration of all simulations to unity and so the unfolding sequence can be directly compared across the entire range of unfolding times and thereby, the entire range of unfolding forces.

**Figure 3:**
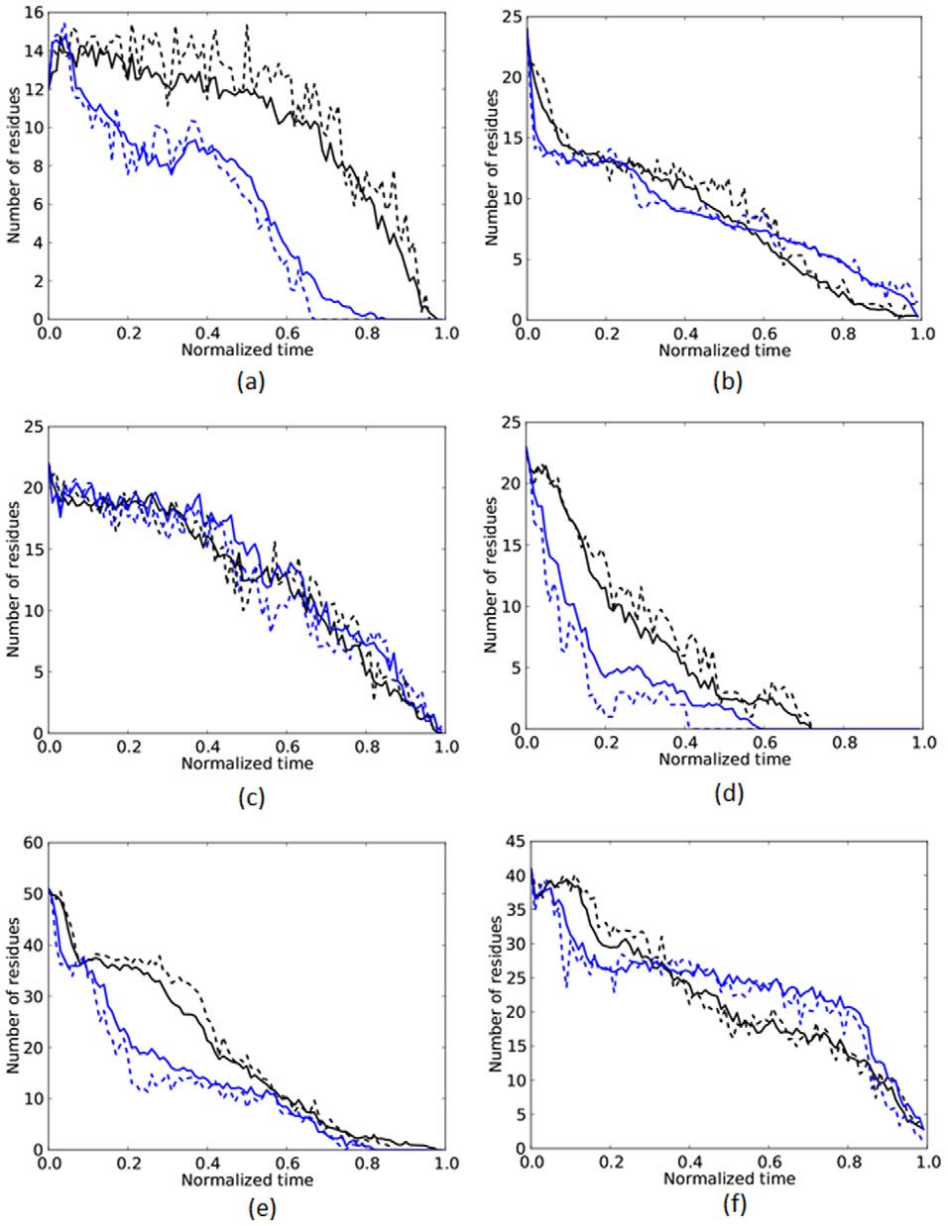
Number of folded 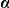-helical (left column) and 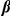 strand (right column) residues as a function of time normalized by the unfolding time. Within each panel: the blue dashed line is at the highest force, 3000 pN; the black dashed line is at the lowest force, 750 pN for barnase and RNase H, or 875 pN for ubiquitin; the blue (black) solid line is an average over a set of the highest (lowest) forces. When viewed versus normalized time, some trajectories, such as the unfolding of barnase's 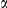 helices and RNase H's 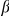 strands, are invariant to force.

Figure 3 compares high- and low-force unfolding of 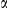 helices (left column) and 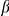 strands (right column). Each panel has four curves of the number of folded residues (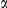 helices or 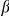 strands) plotted versus nondimensional time. On each panel, the result at the highest force, 3000 pN, is shown as a dashed blue line. Unfolding at the lowest force, 750 pN (barnase and RNase H) or 875 pN (ubiquitin), is shown as the dashed black line. Each of these two curves at the extremal forces represents an average over typically four simulations at a single highest or lowest force. For the remaining two curves, the applied forces were divided into a high- and a low-force set. The solid blue line is the average of all high-force simulations (as listed here in pN– ubiquitin 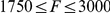; barnase 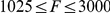; RNase H 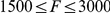). The solid black line is the average of all low-force simulations (ubiquitin 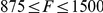; barnase 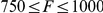; RNase H 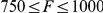).

The extent to which the shape of the dashed curve for the single maximum (minimum) force varies from the solid curve for the set of high (low) forces indicates the extent of variation of the unfolding pathways within the set of high (low) forces.

As can be seen from all panels, there is little change between the unfolding within each high-force (blue) or low-force (black) set. There is, though, a general difference between high and low force unfolding. For example, for ubiquitin and RNase H, 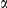 helices unfold faster (measured in normalized time) at high than at low forces, Figure 3 (a) and (e). Strikingly, ubiquitin's 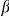 strands and the 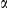 helices of barnase unfold along a similar time history across *all* forces tested, Figs. 3 (b) and (c), respectively.

Of course, the dimensional duration of unfolding is shortened due to its speed-up with applied force. Figure 3 reveals that unfolding trajectories follow similar pathways within a set of high or low forces when viewed in nondimensional time.

### Unfolding times and length scales

It is usual to plot the logarithm of unfolding time 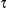 versus applied force 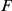, 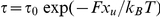, where 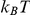 is the thermal energy (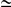
4.1 pN-nm for our simulations), 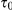 is the inverse of a rate constant, and 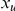 is the change in end-to-end length from the native to the transition state. At the large forces used in this study, 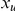 is found to be sub-angstrom, (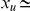
0.002 nm for 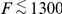 pN). This unphysically small length scale indicates that the exponential scaling is inapplicable at these high forces [37].

**Figure 4:**
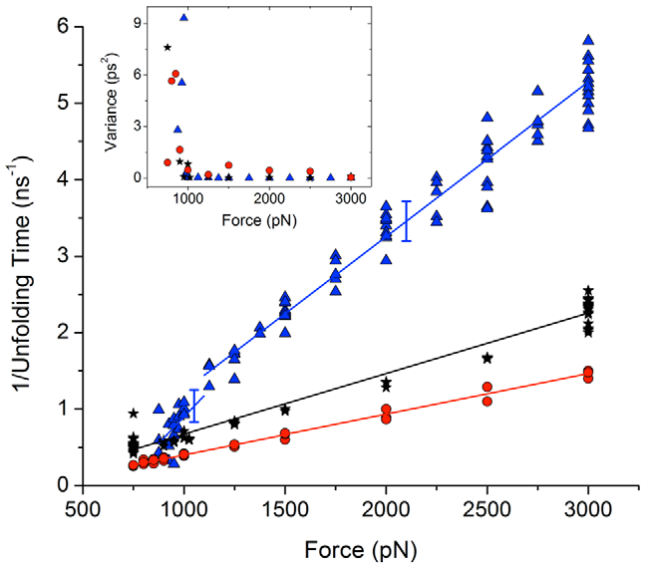
Inverse of unfolding times as a function of force for ubiquitin. Blue 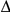; barnase, black 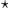; and RNase H, red 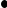. The error bars show the root mean square errors (RMSE) of the linear fits to the ubiquitin data above and below the crossover force. The RMSE for ubiquitin and barnase are comparable. The inset shows the variance for the sets of data at each force. There is a large increase in the variance near the crossover force. The values of variance shown have been scaled by 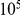, 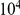, 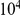, for ubiquitin, barnase and RNase H, respectively, to allow them to presented within the range (0 10) ps^2^.

Alternatively, as seen from Fig. 4, the inverse of the high-force unfolding times is well fit by the line,
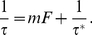
(1)If the slope 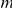 and time scale 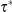 in Eq. 1 are to independently determine a characteristic length and time scale, then dimensional analysis indicates that the units of 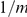 must be force per time. The expectation that this term should be affected by thermal energy suggests 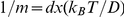, where 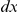 is a length and 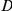 is the diffusivity. Using Einstein's relation, 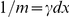, where 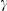 is the viscous drag coefficient. Prior research has emphasized the importance of friction for high-speed and high-force pulling [38]–[40]. These works used the Langevin equation, in which the viscous contribution is a damping coefficient times the velocity. In our study, we consider the geometric contribution to the viscous term. While 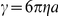 is appropriate for spherical geometries with radius 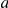, we anticipate that the drag acts along the slender geometry of the drawn-out thread of unfolded residues of length 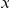. For such a geometry, 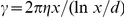, where 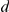 is the diameter of the withdrawn protein, 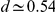 nm [41], [42]. As the protein is pulled, viscous stresses act over a thread of unfolded residues which increases in length from zero to the full unfolded length. To determine 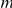, we integrate over the entire unfolding process from zero to full extension, 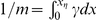, where 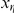 is chosen to fit the measured slope of inverse time versus force as shown in Fig. 4. The values of the viscous length scales 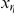 are shown in Table 1. Perfect agreement with measurement would yield 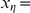
Unfolded length. The values for ubiquitin and RNase H are in excellent agreement. It is interesting that a relationship of the form Eq. 1 is also seen for protein translocation through pores [43], [44]. In that case, as well as here, the relationship is found to hold only at high enough forces.

**Table 1:** Viscous length scales.

	Ubiquitin	Barnase	RNase H
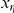	24	44	52
Unfolded length	25	38	52

Length scales in nm. The viscous length scale 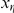, as determined from the best fit of the high force data to Eq. 1, scales with the unfolded length. The values for ubiquitin and RNase H are in excellent agreement. The viscous length for barnase is approximately 16% greater than the maximum possible unfolded length.

For each protein, the 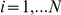 data points 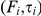 for the unfolding times 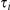 at forces 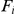 were divided into a low- 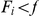 and high-force 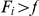 set in which the low- (high-)force set contains from 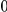 to 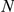 (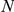 to 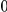) points as 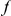 is increased. For each 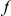, Eq. 1 was fit to the low- and high-force data sets, and the sum of the mean-square error (MSE) from both lines determined. The best fit minimizes the sum of the MSE from both lines over all 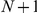 values of 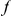. The intersection of the two lines identifies a crossover force between low-and high-force regions. Using the MSE, the crossover is clearly identifiable, at 1100 pN, only for ubiquitin, see Fig. 4. As discussed below, similar crossover values can be identified for barnase and RNase H from the measurements of variance, coefficient of variation, in the variation of front propagation speed, and from the appearance, below a crossover force, of intermediate states and cooperativity.

### Coefficient of Variation

**Figure 5:**
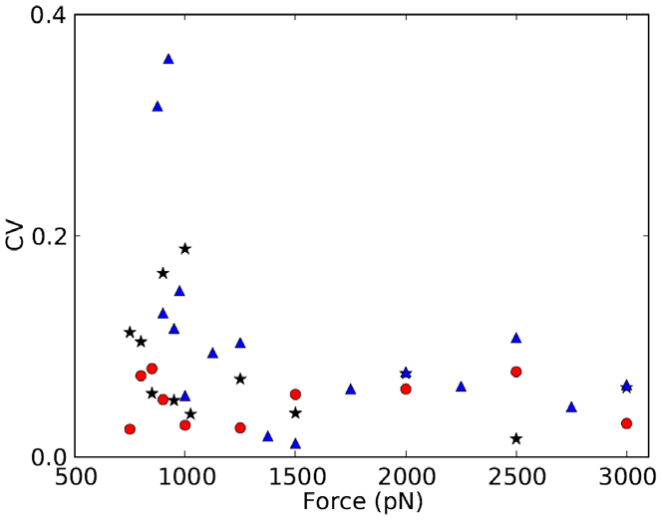
The coefficient of variation (CV) in unfolding times as a function of force for ubiquitin. Blue 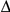; barnase, black 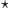; and RNase H, red 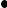. (Barnase values are 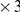.) While RNase H shows little trend, for ubiquitin and barnase CV increases as force decreases, especially below 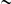
1100 pN, indicating larger fluctuations in the unfolding times at low forces.

The existence of a crossover force can also be inferred for ubiquitin and barnase by using the variance of the unfolding times. The inset to Fig. 4 shows a large increase in the variance of the three proteins near the crossover force. The coefficient of variation (CV) normalizes the variance to adjust for changes in mean values. So, to compare fluctuations in unfolding times around the different mean values at each force, we use the coefficient of variation (CV), which is the square root of the variance divided by the mean, Fig. 5. Thus, CV is the scatter of the data measured as a fraction of the mean; constant CV indicates that variation is a fixed percentage of the mean. A least-squares linear fit to the natural log of CV yields slopes of 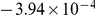 and 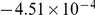, for ubiquitin and barnase, respectively. The CV for RNase H shows little change: the slope of its fit is 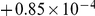. The CV for ubiquitin and barnase increases as force decreases, particularly at forces below 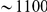 pN, indicating that their unfolding times are more variable at low forces than at high forces.

Alternatively, the ratio of mean square errors measured below the crossover to that above the crossover is found to be much higher than unity, 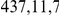 for ubiquitin, barnase, and RNase H, respectively.

### Front propagation speed

As described in the Methods section, the protein is fixed at its N-terminus and the force is applied to the C-terminus. Residues tend to unfold sequentially from the pulling (C-terminus) end. As can be seen in Fig. 1c, the time of the earliest transitions to coil (shown in white) occur at the highest residue number and progress to residue number one. We call this type of sequential unfolding, front propagation, as there is a sharp demarcation or front at the incipiently unfolding residue which separates the remaining folded protein from the string of unfolded residues.

**Figure 6:**
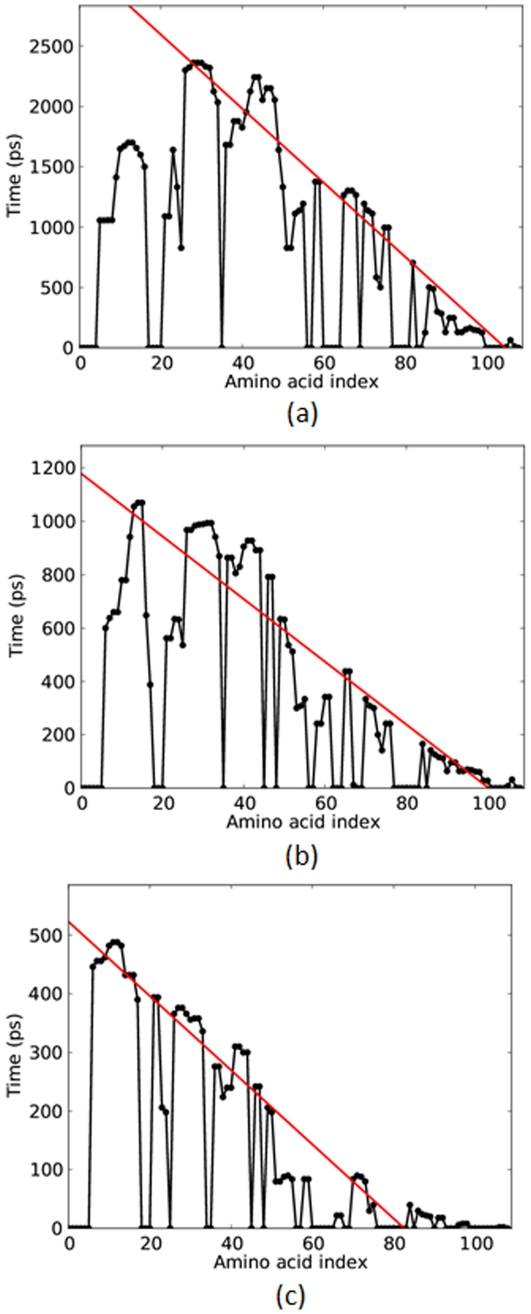
The time of unfolding to coil of each residue of barnase under the applied forces shown. A (red) line is fit to the time of change to the coil state to show the propagation of the unfolding front from the pulling end. The slope of such unfolding fits are collected for the three proteins in Fig. 7. (a) 750 pN, (b) 1500 pN, (c)3000 pN.

From data such as presented in Fig. 1, the time of each amino acid's final transition to the coil state is found, with the coil state as defined by [36]. A set of points with coordinates (residue number, transition time to coil) is generated, Fig. 6. An upper envelope, which covers the original data, is then composed of only those points whose transition time to coil is a local maximum. The global maximum, that is, the time at which the last secondary structure makes the transition to the coil state, is also determined. As shown in Fig. 6, a best fit line is calculated using the least-squares method through the local maxima up to the global maximum. The inverse of the slope of this line yields the propagation speed of the unfolding front in residues/ps.

**Figure 7:**
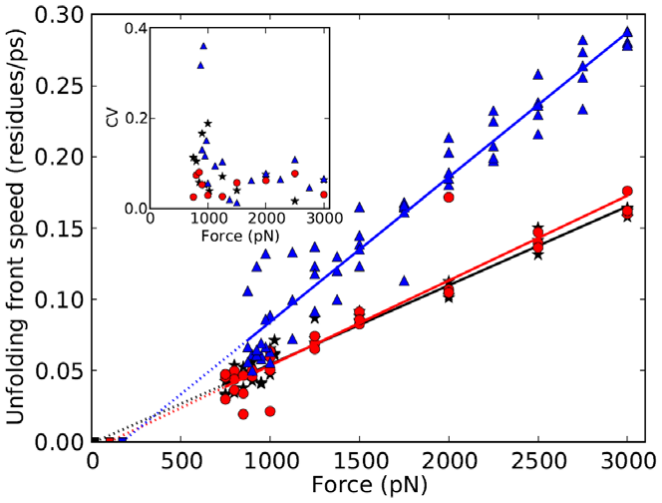
Propagation speed, in residues per ps, for the unfolding front for ubiquitin. Blue 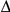; barnase, black 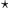; and RNase H, red 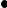. The extrapolation of the best fit lines intercept zero propagation speed between 2 and 100 pN, suggesting that there is a finite force at which the unfolding front is not viable. Inset: Coefficient of variation (CV) of the front propagation speed increases sharply for the three proteins below approximately 1100 pN.

The propagation speed for barnase and RNase H are comparable and approximately one-half that for ubiquitin, Figure 7. For the three proteins, the propagation speed 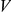 is a linear function of the applied force, 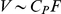, where the protein-dependent 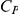 [residues/(ns-pN)] is found to be: 0.10, ubiquitin; 0.06, barnase; 0.06, RNase H.

Taking the change in length on unfolding as the difference between the unfolded length of an average residue, namely 0.363 nm [45], and the projected length of an 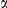 helix residue along the helix axis, namely 0.15 nm [46], then the speeds fall in the range of 6 to 36 m/s. This speed is well below the sound speed in water, which is approximately 1500 m/s, and also less than the sound speed in a polymer with a shear modulus in the GPa range. Comparable propagation speeds, up to 16 m/s were previously reported for unfolding of vimentin at high constant-speed pulling [47]. The data extrapolates to zero propagation speed at a force of approximately 2, 41 and 100 pN for ubiquitin, barnase, and RNase H, respectively, Fig. 7. The finite-valued intercept suggests that the unfolding front does not persist at low enough forces.

The coefficient of variation of the front propagation speed increases markedly below a cutoff force of approximately 1000 pN, as seen in the inset in Fig. 7. The increasing CV indicates the breakdown of front propagation and the appearance of cooperative unfolding as described in the section, Cooperativity, below.

At low forces, there may be a second unfolding front propagating from the fixed (N-terminus) end, Fig. 6, though it is not as well defined as the one propagating from the pulling end. When present, unfolding propagation from the fixed-end commences after a delay with respect to the onset of pulling. The number of residues unfolded from the fixed end is small and decreases to zero as the applied force increases.

## Discussion

A crossover force 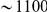 pN, due to a fundamental limiting speed, marks a change in unfolding trajectories. Above the crossover, residues unfold sequentially from the pulling terminus along a front of advancing force whose speed is proportional to the applied force. Stresses originating from the pulling end propagate no further than the residue at the verge of extraction from the remaining unfolded residues. Below the crossover force: (i) plateau regions of constant extension indicating intermediate states appear, Figs. 1 and 2, (ii) non-sequential unfolding occurs, in which certain residues do not unfold at the unfolding front, Fig. 1, rather (iii) the unfolding of key residues is correlated with the onset and termination of these intermediate states. These features of the unfolding pathway are as seen in measurements with forces lower by one or two orders of magnitude. We find the same intermediate states as seen in AFM studies. The same specific residues which unfold cooperatively at forces of O(10) pN are seen at high forces, unfolding following the low-force sequence. These critical cooperative events are thus found to be insensitive to force.

The expression 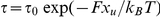 has the geometric representation of tilting the zero-force energy profile by 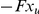. However, the unphysical values found for 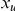 indicate that tilting is not the correct interpretation at high forces, as also supported by the presence of cooperativity and intermediate states. As suggested by theories which compare unfolding to nucleation processes [48], the strength of cooperative structures arises from the unfolding fragments being significantly different from the folded cooperative structure. Unfolding, persists at high force while awaiting a large-enough fluctuation. This finding suggests how native structures are designed to be cooperative, to resist high transient forces, and to provide strength at *all* forces. It further hints at means to design man-made protein structures for high strength [49].

Solvent viscosity slows protein motion. Protein unfolding takes place at low Reynolds number 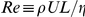, where 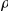 is solvent density, 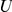 is a characteristic speed of motion, 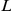 is a characteristic size of the part that is moving, and 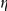 is the dynamic viscosity, which for water is approximately 0.89 pN-ns/nm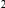 at room temperature [50]. The small size of proteins yields 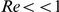. At these low Reynolds numbers, a well-known result from fluid mechanics is that viscous forces can be written as the product of three factors, 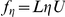, where 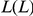 depends *only* on the geometry of the moving object [50]. The best known example is the so-called Stokes drag for a sphere, 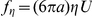, where 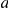 is the radius. In general, viscous forces are difficult to measure due to slow speed and small size, which multiply to yield a small value of the viscous force. The large applied forces used here, lead to relatively large speeds of unfolding, making viscous force the controlling aspect of unfolding. Equation 1 for the unfolding time can be recast as 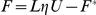, where 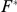 is a constant and 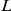 is the geometric factor appropriate for the unfolding chain of residues. Using this expression, we show the validity of the continuum hydrodynamics geometric factor 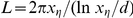.

The viscous forces in the high-force regime are easy to measure by comparison with the unfolding time as a function of force, shown in Fig. 4. Viscous drag for similar geometries 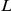 can be precisely extrapolated by using the analytic scaling 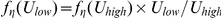, to find the hydrodynamic drag at low speeds 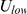 expected at low applied force. It would be exceedingly difficult to have made these measurements, with either physical experiments or numerical simulations, at low speeds.

The high forces used here are not encountered *in vivo*. However, our goal was not to reproduce the physiologic environment, but to reveal with high forces that which is difficult or impossible to perceive with low-force methods. Recall that directly attempting all-atom explicit-solvent simulations at low forces would have led to prohibitively long run times. Using high force, we find that the sequence of cooperative unfolding remains invariant up to 1100 pN or more. Using scaling relationships [51], lifetimes of these cooperative structures at physiologic forces can now be determined. Similarly, high forces provide large enough speeds such that the form of the viscous stress can be readily determined, and hydrodynamic theory provides the scaling relationship to extrapolate these findings to low force measurements. Though the full extent to which unfolding at extremely high forces reproduces aspects of cooperative unfolding at forces in the range of 10's of piconewtons awaits further measurements, this study provides preliminary validation that high applied forces may be useful to accelerate all-atom explicit-solvent molecular dynamics simulations such that they span the entire unfolding process while allowing a detailed view of persistent cooperative events along the unfolding pathway. Unfolding under physiologic-level forces may occur over a time course too long to be simulated. Or, even if possible, lengthy simulation time may preclude taking a sufficient sampling of runs to determine statistical properties or determine mean expectations. High-force simulations run faster and allow repeated simulations within a feasible time.

## Methods

**Figure 8:**
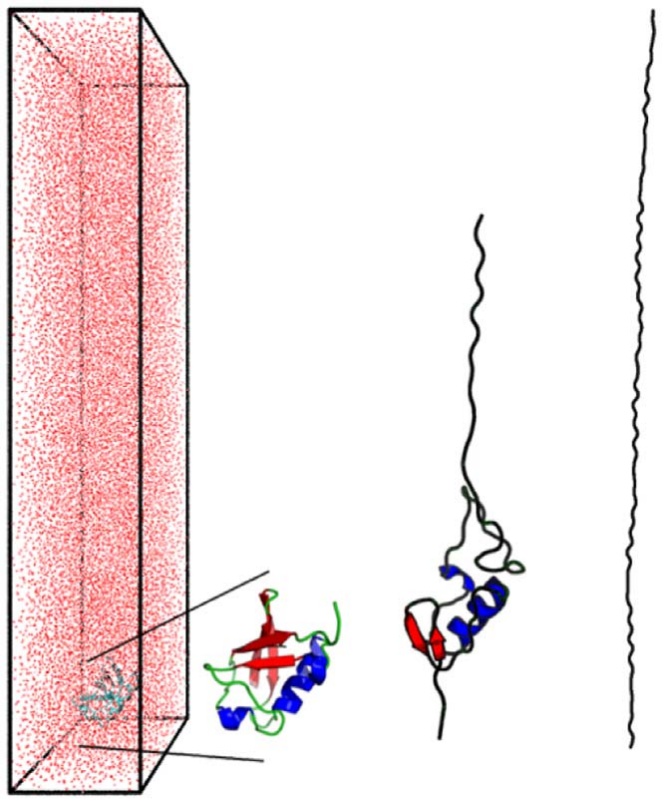
Molecular dynamics simulations. The simulation volume contains at least 73,000 water molecules, appearing here as a reddish haze surrounding the protein ubiquitin in its native structure as obtained from the Protein Data Bank. Snapshots from the MD simulation show the initial structure enlarged, along with a partially unfolded and nearly fully-stretched ubiquitin.

All-atom explicit-solvent molecular dynamics used the GROMACS 4.0.5 [52] simulation package with OPLS-AA force field [53], [54] and a time step of 2 femtoseconds, Fig. 8. The protein was initialized in its native state, as determined from its Protein Data Bank [55] structure and subsequently unfolded by applying a constant force at the C-terminus while the N-terminus was held fixed. Applied forces ranged from 750 to 3000 pN. The force was applied on each C, N, and O atom in the C-terminus residue, and similarly each heavy atom of the N-terminus was frozen in place to establish the fixed-end boundary condition.

The three proteins used in this study present contrasting structural forms: ubiquitin has a long central 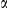 helix flanked by 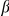 sheets; barnase is highly 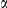 helical over its N-terminus half, and is 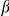 strand rich for its second half; RNase H is largely 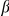 sheet, followed by five 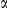 helices [56]–[58]. For these proteins, folding and unfolding pathways have been extensively investigated numerically and experimentally [11], [30], [32]–[34], [59]–[66].

Three to five simulations were completed for each protein at each applied force. In order to calculate distributions of unfolding times at a given force, three cases were chosen for additional study: a total of 30 simulations were run for ubiquitin at 3000 (pN), 20 for barnase at 3000 pN, and 20 for barnase at 750 pN. The initial conformation was placed near the bottom of an elongated water box with periodic boundary conditions. The size of the simulation cell varies between proteins, and is minimized such that it can contain the fully-extended molecule, and that the protein does not cross any boundaries in its initial state or during unfolding, Table 2.

**Table 2:** Model details.

	(w, h, d)	Protein atoms	Solvent atoms	ns/day
Ubiquitin (1UBQ)	(5, 30, 5)	1231	73116	8.7
Barnase (1BNR)	(5, 45, 5)	1727	159840	4.8
RNase H (1RCH)	(6, 60, 6)	2455	212709	2.0

Width (w), height (h) and depth (d) of the simulation volume in nm, the number of protein atoms, the number of water molecules, and typical performance in nanoseconds of simulation time per day, for each of the three proteins.

Water molecules are modeled explicitly according to the TIP3P model [67]. The number of water molecules depends on the simulation box size and the protein, but is never less than 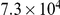, Table 2. After adding water molecules to the simulation box, energy minimization is applied using the method of steepest descent for 5,000 steps, and the system is equilibrated for 100 ps. To ensure consistency, the same minimized and equilibrated configuration is used as the starting point for all simulations of a given protein. As energy is introduced to the system through the applied force, the Berendsen thermostat is used to maintain a temperature of 298 K.
